# The Tropical Invasive Seagrass, *Halophila stipulacea*, Has a Superior Ability to Tolerate Dynamic Changes in Salinity Levels Compared to Its Freshwater Relative, *Vallisneria americana*

**DOI:** 10.3389/fpls.2018.00950

**Published:** 2018-07-04

**Authors:** Michelle A. Oscar, Simon Barak, Gidon Winters

**Affiliations:** ^1^French Associates Institute for Agriculture and Biotechnology of Drylands, Jacob Blaustein Institutes for Desert Research, Ben-Gurion University of the Negev, Midreshet Ben-Gurion, Israel; ^2^Dead-Sea & Arava Science Center, Neve Zohar, Israel

**Keywords:** seagrass, *Halophila stipulacea*, salinity, *Vallisneria americana*, seagrass physiology, aquatic plants physiology, photochemistry, invasive seagrass

## Abstract

The tropical seagrass species, *Halophila stipulacea*, originated from the Indian Ocean and the Red Sea, subsequently invading the Mediterranean and has recently established itself in the Caribbean Sea. Due to its invasive nature, there is growing interest in understanding this species’ capacity to adapt to new conditions. One approach to understanding the natural tolerance of a plant is to compare the tolerant species with a closely related non-tolerant species. We compared the physiological responses of *H. stipulacea* exposed to different salinities, with that of its nearest freshwater relative, *Vallisneria americana*. To achieve this goal, *H. stipulacea* and *V*. *americana* plants were grown in dedicated microcosms, and exposed to the following salt regimes: (i) *H. stipulacea*: control (40 PSU, practical salinity units), hyposalinity (25 PSU) and hypersalinity (60 PSU) for 3 weeks followed by a 4-week recovery phase (back to 40 PSU); (ii) *V*. *americana*: control (1 PSU), and hypersalinity (12 PSU) for 3 weeks, followed by a 4-week recovery phase (back to 1 PSU). In *H. stipulacea*, leaf number and chlorophyll content showed no significant differences between control plants and plants under hypo and hypersalinities, but a significant decrease in leaf area under hypersalinity was observed. In addition, compared with control plants, *H. stipulacea* plants exposed to hypo and hypersalinity were found to have reduced below-ground biomass and C/N ratios, suggesting changes in the allocation of resources in response to both stresses. There was no significant effect of hypo/hypersalinity on dark-adapted quantum yield of photosystem II (*F*_v_/*F*_m_) suggesting that *H. stipulacea* photochemistry is resilient to hypo/hypersalinity stress. In contrast to the seagrass, *V. americana* exposed to hypersalinity displayed significant decreases in above-ground biomass, shoot number, leaf number, blade length and *F*_v_/*F*_m_, followed by significant recoveries of all these parameters upon return of the plants to non-saline control conditions. These data suggest that *H. stipulacea* shows remarkable tolerance to both hypo and hypersalinity. Resilience to a relatively wide range of salinities may be one of the traits explaining the invasive nature of this species in the Mediterranean and Caribbean Seas.

## Introduction

Seagrasses (order: Alismatales) are a unique group of marine flowering plants (angiosperms) that re-entered the oceans and secondarily colonized marine habitats. Phylogenetic analysis of the Alismatales has indicated that the secondary return of seagrasses to the sea occurred three–four times independently through parallel evolution from a common aquatic (freshwater) ancestor of terrestrial origin (Les et al., 1997; Orth et al., 2006; Wissler et al., 2011). In returning to the sea, some 60–90 million years ago (Les et al., 1997), seagrasses were faced with the physiological challenges related to growing in marine conditions (Olsen et al., 2016). These challenges entailed adaptive morphological and physiological traits common across all seagrass species, causing them to be considered as a single functional group (Les et al., 1997; Olsen et al., 2016). These adaptive traits include blade or subovate leaves, epidermal chloroplasts, loss of the stomata, internal gas transport that facilitates root proliferation in permanently flooded anoxic sediments (den Hartog, 1970; Hemminga and Duarte, 2000), submarine (hydrophilous) pollination (Björk et al., 2008) and tolerance to high salinity environments (Touchette, 2007).

*Halophila stipulacea* (Forsk.) Aschers. is a small, dioecious tropical seagrass that was originally described from the Red Sea (Forskal, 1775; Lipkin, 1975). It is widely known as a euryhaline species because of its wide range of salinity tolerance (den Hartog, 1970; Por, 1971). *H. stipulacea* was first reported in the Mediterranean Sea in 1894, only 25 years after the opening of the Suez Canal, making it a Lessepsian migrant (Lipkin, 1975). Since then it has become established along the coastlines of Egypt, Lebanon, Turkey, Albania, Greece, Italy, Libya, Cyprus and Tunisia (Lipkin, 1975; Gambi et al., 2009; Sghaier et al., 2011). Surprisingly, in 2002, this seagrass was reported in the Caribbean Sea (Ruiz and Ballantine, 2004), and in just over 10 years has spread to most eastern Caribbean island nations and recently to the South American continent (Vera et al., 2014; Willette et al., 2014; van Tussenbroek et al., 2016). Studies from the Caribbean have shown that *H. stipulacea* is physically displacing local Caribbean seagrass species (e.g., *Syringodium filiforme*) by monopolizing their space (Willette and Ambrose, 2012; Steiner and Willette, 2014). It has been suggested that the invasiveness of *H. stipulacea* could be attributed to its ability to acclimate to a wide range of physiological conditions including water temperatures, light intensities, nutrient levels and salinities (Por, 1971; reviewed by Gambi et al., 2009; Sharon et al., 2009, 2011).

Salinity is a major environmental component that can influence the growth, function, structure, and distribution of seagrasses (Montague and Ley, 1993; Salo et al., 2014). Most seagrass species are adapted to grow at salinities ranging from 20 to 40 Practical Salinity Units (PSU; 35 PSU having a concentration for the major ions of 540 mM Cl^-^ 460 mM Na^+^, and 50 mM Mg^+^; Touchette, 2007). While these salt concentrations are much higher than those found in saline habitats where terrestrial plants exist, changing salinities can also influence the structure and function of seagrass communities (Montague and Ley, 1993), including the disappearance of seagrass meadows (Zieman et al., 1999; Rudnick et al., 2005).

In terms of their ability to withstand marine salinities, seagrasses are halophytes, i.e., they can thrive in salt concentrations that would kill 99% of other plant species (Flowers and Colmer, 2008). Increases or decreases in salinity have been shown to affect the photosynthetic capacity, growth, pigment content, biomass and C/N balance in seagrasses (Fernández-Torquemada and Sánchez-Lizaso, 2011; Sandoval-Gil et al., 2012; Garrote-Moreno et al., 2015; Piro et al., 2015a,b). Several studies have been conducted to study salt tolerance in both temperate and tropical seagrasses (Koch et al., 2007; reviewed by Touchette, 2007; Sandoval-Gil et al., 2014; Collier et al., 2014). Koch et al. (2007) tested the response of three tropical seagrass species to hypersalinity (induced slowly and also pulsed). Based on growth and photosynthetic parameters, they demonstrated that *Thalassia testudinum* and *Halodule wrightii* are able to tolerate high salinities of 60 and 65 PSU, respectively. Interestingly, Sandoval-Gil et al. (2014) reported that inter and intra-specific divergences play an important role in determining the threshold of salinity tolerance in the temperate seagrasses *Cymodocea nodosa* and *Posidonia oceanica*. This is indicated by the intra-specific physiological plasticity that was observed between different populations of *Cymodocea nodosa* and *P. oceanica*. While Collier et al. (2014) also noticed inter-species specific hyposalinity thresholds, they proposed a response analogous to a stress-induced morphometric response (SIMR) – the seagrasses show up to a 400% increase in shoot density at sub-lethal salinities. This SIMR precedes mortality in two of the three seagrass species chosen for the study (Collier et al., 2014).

The only known study investigating salinity tolerance in *H. stipulacea* has shown that the epidermal concentrations of Na^+^ and Cl^-^ are lower than the surrounding seawater, indicating the existence of some ion exclusion mechanisms (Beer et al., 1980). They also demonstrated that carbon-fixing enzymes such as phosphoenolpyruvate carboxylase are able to function in the presence of salt *in vitro*, an important adaptive mechanism to salinity. However, very few studies have measured responses of tropical seagrasses to both hyper and hyposalinities (Collier et al., 2014), and none have compared responses of seagrasses to differing salinities with that of their freshwater relatives. Comparing close halophytic and non-halophytic terrestrial relatives has proven a very fruitful approach to understanding halophytism in the *Brassicaceae* (Inan et al., 2004; Amtmann, 2009; Orsini et al., 2010; Oh et al., 2012; Bartels and Dinakar, 2013). These comparative studies have revealed a plethora of salt adaptation mechanisms including anatomical structures, tight control of entry and compartmentation of Na^+^ uptake, salt-resilient photochemistry, constitutive up- and down-regulation of stress tolerance genes and metabolites, and sub-functionalization and neo-functionalization of duplicated genes (Inan et al., 2004; Volkov et al., 2004; Kant et al., 2006; Stepien and Johnson, 2009; Dassanayake et al., 2011; Kazachkova et al., 2013; Oh et al., 2014). Similarly, the recent comparison of the *Zostera marina* genome with a freshwater relative (the duckweed, *Spirodela polyrhiza*) has revealed genetic changes related to adaptation to a marine environment (Olsen et al., 2016).

The order of *Alismatales* includes eleven families of aquatic freshwater species and four families that are fully marine species (i.e., seagrasses; Les et al., 1997; Wissler et al., 2011). The phylogenetically closest freshwater relative of *H. stipulacea*, the tropical seagrass species which is the focus of our study, is *Vallisneria americana* (Les et al., 1997). Both species belong to the *Hydrocharitaceae* family, and both the *Halophila* and *Vallisneria* genera are classified within the same subfamily (Hydrilloideae Luerss.; reviewed by Les et al., 2006). In fact, phylogenetic studies of the Hydrocharitaceae that compared both morphological characteristics and a suite of molecular markers (chloroplast rbcL, matK, trnK intron sequences, ribosomal ITS region sequences; Les et al., 2006), showed that *H. stipulacea* is more closely related to *V*. *americana* than to many other seagrass species, and the two species share some metabolic features such as production, accumulation, and use of carbohydrates (Kraemer et al., 1999). Although *V. americana* is considered a freshwater (aquatic) angiosperm (Kraemer et al., 1999), several studies suggest that *V. americana* can tolerate up to 20 PSU (Doering et al., 1999; Boustany et al., 2010). However, the salinity tolerance limits of *V. americana* varies between populations (Doering et al., 1999; Frazer et al., 2006; Boustany et al., 2010; Lauer et al., 2011).

Applying the comparative approach used to understanding salt tolerance mechanisms in terrestrial halophytes, the aim of this study was to lay the foundations for detailed molecular investigations into *H. stipulacea* halophytism by comparing the physiological responses to changes in salinity in the tropical seagrass *H. stipulacea*, with that of its closest freshwater relative, *V. americana*. To the best of our knowledge, this is the first comparison of physiological and growth responses of a seagrass species with its freshwater relative.

## Materials and Methods

### Plant Collection and Experimental Design

**FIGURE 1:**
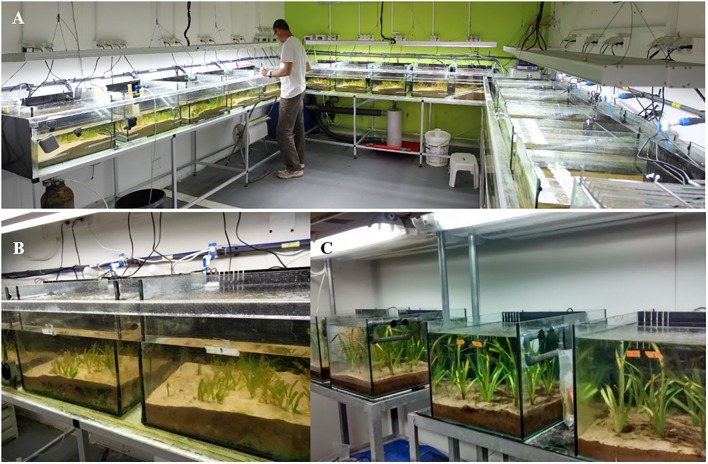
**(A)** The seagrass (*Halophila stipulacea*) dedicated microcosm fully controlled for water temperature, light, and salinity (the image is being published with the consent of the depicted individual; Photographed by Yoram Zvieli). **(B)** *H. stipulacea* growing in some of the aquaria (photographed by Gidon Winters). **(C)** The *Vallisneria americana* dedicated microcosm with aquaria and sediment type identical to those used in the seagrass setup (photographed by Gidon Winters).

Intact *H. stipulacea* plants were collected from meadows growing at 6–8 m depth (Supplementary Figure S1) in the northern Gulf of Aqaba (North beach site; 29.546150° N 34.964819° E; Mejia et al., 2016) by SCUBA-diving. In order to reduce variability, only shoots less than 1 year-old were collected (i.e., shoots with less than 12 leaf scars on the vertical rhizome; Pérez and Romero, 1992). Plants were brought into our seagrass dedicated microcosm with controlled temperature, salinity and light (**Figure 1A**) and planted in 15 aquaria (40 cm width × 33 cm height, ∼45 L of seawater in each aquarium), placed in temperature-controlled water baths at 25°C and layered with 20 L of natural sediment (10 cm high; **Figures 1A,B**). Two months prior to collecting plants, sediment was collected from a location near the plant collection site, sieved (∼3 mm pore) to exclude macro-invertebrates and stones, autoclaved (to exclude potential microbial contaminations) and placed into aquaria. In each aquarium, 13 shoots (with their corresponding rhizomes and roots) were planted in 10 cm of sediment and the aquarium was filled with artificial seawater (Red Sea Salt, Israel) at a control salinity level of 40 PSU (the year-round average salinity of Eilat’s water^1^). Lighting was provided via T5 fluorescent lamps (Osram Lumilux HO 865/54W cool daylight with the color temperature of 6500 degrees Kelvin^2^). Photosynthetically active radiation (PAR) values and duration (10–12 h of light, ∼100–120 μmol photons s^-1^ m^-2^) at the bottom of the aquaria were set to mimic midday light at the site during November 2016 at 6–8 m depth. Independent powerheads were installed to obtain proper water circulation in each aquarium. Salinity, pH, and temperature were measured in each aquarium daily using a digital salinity/conductivity/temperature meter (WTW 340i, WTW, Germany). Conditions (25°C, 40 PSU) in the microcosms were maintained for 21 days to allow plant acclimation before starting experiments.

**FIGURE 2:**
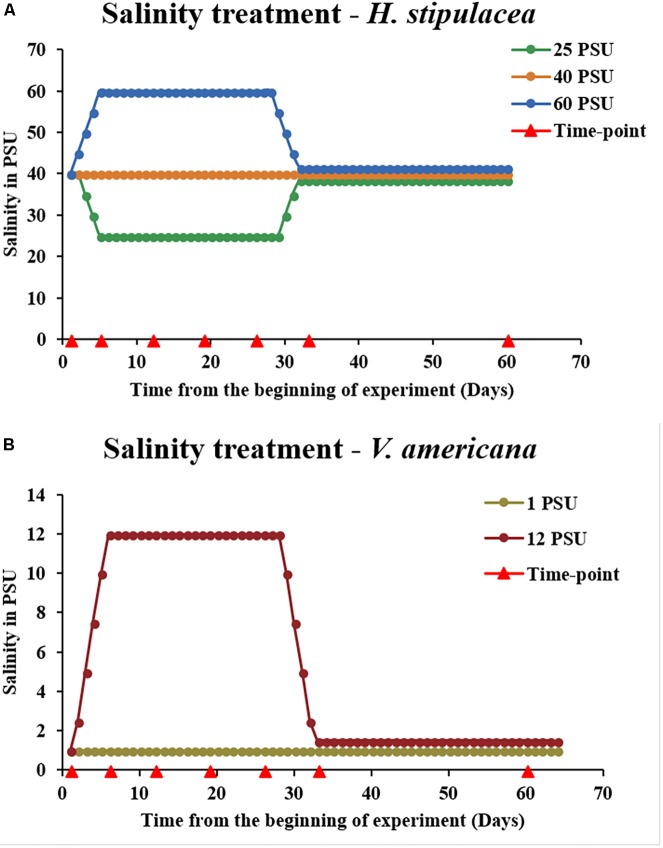
Experimental design for investigating the physiological response of *H. stipulacea* **(A)** to control, hyper and/or hyposalinity (40, 60, and 25 PSU, respectively) and its freshwater relative, *V. americana* **(B)** to control and hypersalinity (1 and 12 PSU, respectively). In both species, there were five tanks for each treatment (*n* = 5). Salinities were kept at these levels for 3 weeks followed by a recovery phase of another 4 weeks (salinities returned to control levels of 40 and 1 PSU, for *H. stipulacea* and *V. americana*, respectively). Physiological, fitness and photosynthetic parameters were performed throughout the experiment at various time-points (red arrowheads). Graphical depiction of salinity levels during the recovery phase in **(A)** (25 and 60 PSU) and **(B)** (12 PSU) were slightly modified for visual clarification by separation of the lines.

For gradual ramping of salinities up from 40 to 60 PSU (5 PSU/day), artificial Red Sea salt mixtures (which also contained a complete set of micronutrients and appropriate salts but negligible nitrates or phosphates^3^) were dissolved in distilled water and added daily to the aquariums in parallel to replacing 2–4 liters of aquarium water until the desired final salt concentrations in the aquariums were reached (**Figure 2A**). Similarly, for the gradual ramping down from 40 to 25 PSU, aquarium water that was set at 40 PSU (acclimation period) was gradually replaced by adding small amounts (2–4 liters/day) of distilled water (0 PSU) until the desired final salt concentration (25 PSU) in the aquaria was reached. This ramping up/down process has been previously shown to be slow enough to prevent (or at least attenuate) osmotic shock (Kahn and Durako, 2006; Griffin and Durako, 2012). A set of control plants (*n* = 5 aquaria) were maintained at 40 PSU throughout the entire experiment (**Figure 2A**). In all three treatments, throughout the entire period of the experiment, 10% of the water (25, 40, and 60 PSU) was replaced each week. The plants were maintained under these conditions for 22 days, and harvested at various time points (**Figure 2A**). Salinities in the aquaria were then ramped either up (from the 25 PSU hyposalinity treatment) or down (from the 60 PSU hypersalinity treatment) to the control salinity (40 PSU) at the same rate and method as at the beginning of the experiment (5 PSU/day). After returning to control salinities of 40 PSU, the plants were allowed to recover at this salinity level over a period of another 21 days and then harvested for analysis. To avoid experimental bias, all tanks and treatments were completely randomized.

Throughout the entire experiment, plants were measured and sampled for a suite of fitness, physiological and molecular parameters. Measurements were taken at the following time points: day 0 – just before starting the salinity treatments; day 5 – the day on which the final salinity concentrations were reached; day 12, day 19, day 26 – at 1-week intervals (when plants were exposed to 1, 2, and 3 weeks, respectively, of high/low salinities); day 33 – 1 day after ramping up/down back to the control 40 PSU for recovery; day 60 – after 4 weeks of recovery at control salinity of 40 PSU (**Figure 2A**). Plant harvesting was always performed at the same time of day (11:30–12:30) in order to take into account any effects of the circadian clock. All growth and physiological measurements were carried out on the second youngest leaf pair, which exhibits the least variability within the shoots and is the most representative tissue (Durako and Kunzelman, 2002).

For the parallel *V. americana* experiments, plants were obtained from a commercial supplier in Israel^4^ in October 2016, after which they were grown in sediment, light, temperature and water circulation conditions similar to the *H. stipulacea* plants (**Figure 1C**).

Preliminary work (with other plants) tested the effects of different salinities on *H. stipulacea* (15, 25, 40, 60, and 65 PSU; Supplementary Figure S2). The fact that previous studies determining salinity thresholds in *V. americana* used different populations and varying durations of stress, frequency, and intensities (Boustany et al., 2015), made it difficult to compare across different salinity stress studies. Hence, preliminary experiments were conducted to test the highest tolerance threshold for *V. americana* (0, 10, 12, and 15 PSU; Supplementary Figure S3). The results of these experiments (Supplementary Figure S3) confirmed the work of Twilley and Barko (1990) showing the highest tolerance for *V. americana* to be around 12–13 PSU. Thus, for *V. americana*, we selected 12 PSU as the higher salinity concentration to stress the plants as the plants died at 15 PSU and there were no significant effects of hypersalinity at 10 PSU (Supplementary Figure S3). Control levels were chosen as 1 PSU (as opposed to 0 PSU), due to the fact that *V. americana* is mostly found growing in estuary water, where natural minimal salinities are larger than 0 PSU (Twilley and Barko, 1990; Kraemer et al., 1999).

Similarly, for *H. stipulacea* we selected 25 and 60 PSU since lower salinity (15 PSU) prevented any recovery of leaf number and *F*_v_/*F*_m_ (Supplementary Figures S2A,C). The hypersalinity treatment of 60 PSU was chosen since our preliminary results showed that going up to 65 PSU seemed lethal to *H. stipulacea* (Supplementary Figure S2).

The idea of the comparison between *H. stipulacea* and *V. americana* was to compare plants exposed to salinities that yielded a response to salt but were not lethal. Hence, the final salinity conditions selected for the *V. americana* experiments were 1 PSU (control) and 12 PSU (hypersalinity). Starting from control levels, salinity was ramped up to 12 PSU (**Figure 2B**) at 2.5 PSU/day (by replacing freshwater with saline mixtures as described above for *H. stipulacea*) to avoid osmotic shock. Measurements were taken at the same time points as for *H. stipulacea* on the leaves from the second youngest shoot.

### Growth and Physiological Measurements

Growth and biochemical parameters including leaf number, leaf area, dry weight of above/below-ground tissues, chlorophyll, and carotenoid content, and carbon/nitrogen ratios in above/below-ground tissues were measured over the course of the experiment as physiological indicators of stress. For leaf and shoot counts, three plants were selected randomly per aquarium, plants were marked, and the number of leaves and shoots were counted for the same three plants at every time-point; these three measurements were averaged into a single biological replicate, and this was performed for five tanks (*n* = 5) in each condition (control, hypo, and hypersalinity). Leaf area was measured by selecting the second youngest leaves from three random plants from each aquarium (at each time point these three measurements were averaged into a single biological replicate, *n* = 5 aquaria in each treatment), and digitally scanned (Cannon Lide 110 scanner). The leaf area was then measured using ImageJ (Abramoff et al., 2004; Mejia et al., 2016). Because the morphology of *V. americana* differs from that of *H. stipulacea* (**Figures 1B,C**), leaf counts were determined as for *H. stipulacea* except that we also measured the number of leaves per shoot (there are more leaves per shoot for *V. americana* than in *H. stipulacea*). Additionally, blade length was measured as indicated in Doering et al. (1999). The three measurements from the same aquarium were averaged into a single biological replicate, *n* = 5 aquaria in each treatment.

### Biomass and C/N Ratios

For biomass and C/N ratios, three plants were randomly selected per aquarium at the end of the experiment (day 60 only, due to the need to sacrifice the entire plant for this measurement) and dried in the oven at 60°C for 24 h. The above- and below-ground tissues were then weighed to compare biomass across the three treatments. For determining C/N ratios, the dried tissues were later ground to powder in a TissueLyser (Retsch GmbH & Co. KG) and 6 mg of the tissue was weighed in a tin capsule and analyzed in a Flash 2000 Organic Elemental Analyzer (Thermo Scientific, USA). Calibration was performed using 2–3 mg of 2,5-Bis(5-tert-butyl-benzoxazol-2-yl)thiophene (BBOT) containing 6.51% N, 72.53% C, 6.09% H, and 7.44% S. Each sample was run for 660 s and was recalculated with a blank (empty capsule) and BBOT standard measurements.

### Chlorophyll Florescence and Pigment Content

For determination of quantum efficiency of photosystem II (*F*_v_/*F*_m_; Genty et al., 1989), five plants were selected per aquarium and fluorescence was measured using a PAM (Pulse Amplitude Modulation) chlorophyll fluorometer (PAM-2500, Walz, Germany). The same five plants were measured over time (at every time point) for any change in quantum efficiency. Measurements from the same aquarium were averaged into a single biological replicate, with *n* = 5 aquaria in each treatment. Leaves were dark adapted for 10 s prior to the measurement using a dark adaption leaf clip (Winters et al., 2011). For measuring chlorophyll and carotenoids, 100 mg fresh tissue was harvested from three plants in each aquarium at each time point. Pigments were extracted in 100% methanol, kept overnight at 4°C in the dark, and measured according to Lichtenthaler (1987) in a microplate reader (EPOCH 2, Biotek Instruments, Inc., United States) according to Warren (2008). Concentrations were normalized to the fresh weight (FW) of the sampled tissues.

### Statistical Analysis

For *H. stipulacea*, a one-way ANOVA with repeated measures (IBM SPSS version 19) with a Greenhouse-Geisser correction was performed to identify the effects of each salinity treatment on the physiological data such as leaf number, leaf area, shoot number, blade length, quantum yield, and chlorophyll content (treated as dependent variables) for measurements at different time-points. *Post hoc* mean comparisons with the Tukey–Kramer HSD test were performed to identify specific salinity or time points which exhibited significant differences. For measurements of biomass and C/N ratios that were taken only at the last time point (day 60), a one-way ANOVA (SPSS version 19) was performed. Since the *V. americana* experiment, included only control (1 PSU) and hypersalinity (12 PSU), pairwise comparisons were performed (control vs. hypersalinity) to find the specific time point which displayed significant differences. All the treatment effects were considered statistically significant at *P* < 0.05. Normality of data was tested using Shapiro–Wilk’s test and QQ plots. Homogeneity of variances was tested using the Levene’s test.

## Results

### Changes in Salinity Have No Effect on *H. stipulacea*’s Capability to Produce New Leaves While *V. americana* Exhibits Growth Reduction and Recovery

**FIGURE 3:**
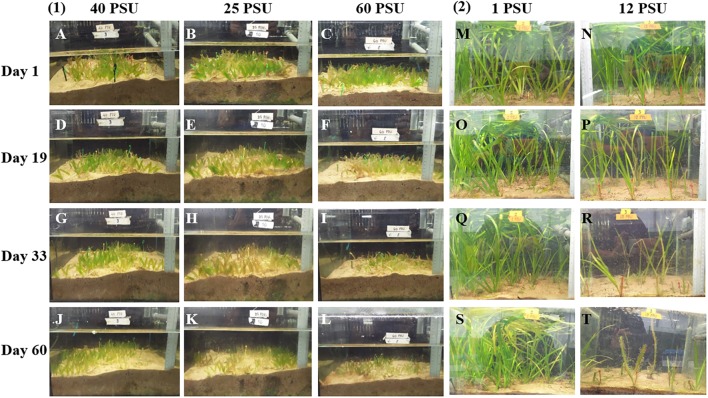
Photographs showing the differences in growth of *H. stipulacea* **(A–L)** and *V. americana* **(M–T)** plants after treatment with different salinities. Days 1–60 correspond to time points during the experiment (see **Figure 2**). **(1)** showing responses of *H. stipulacea* plants to control (40 PSU; **A,D,G,J**), hyposalinity (25 PSU; **B,E,H,K**) and hypersalinity (60 PSU; **C,F,I,L**). **(2)** showing responses of *V. americana* plants to control (1 PSU; **M,O,Q,S**) and hypersalinity (12 PSU; **N,P,R,T**).

In order to examine the effect of hypo and hypersalinity on the growth of the tropical seagrass, *H. stipulacea* and its closest freshwater relative, *V. americana*, the seagrass was exposed to 40, 25, and 60 PSU (control, hyposalinity, and hypersalinity treatments, respectively) while *V. americana* was exposed to 1 and 12 PSU (control and hypersalinity treatments, respectively). **Figure 3** shows images of *H. stipulacea* and *V. americana* plants at various time points (**Figure 2**) during the experiment.

### Leaf Number

**FIGURE 4:**
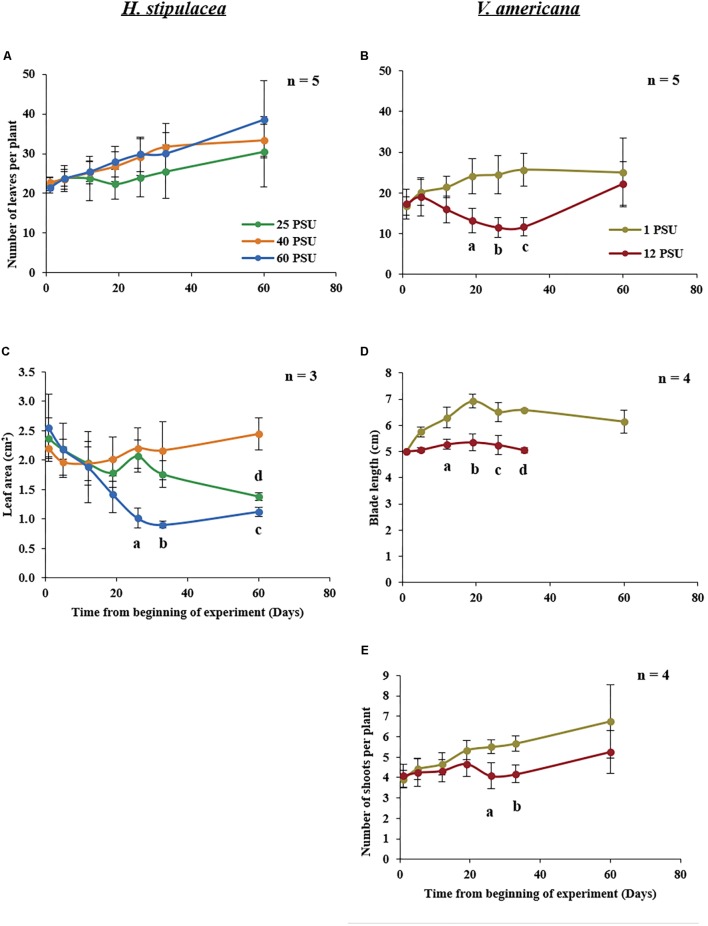
Growth measurements in *H. stipulacea* (Left; **A,C**) and *V. americana* (Right; **B,D,E**) throughout the experiment. *H. stipulacea* plants were exposed to control, hyper and hyposalinity (40, 60, and 25 PSU, respectively). *V. americana* plants were exposed to control and hypersalinity (1 and 12 PSU, respectively). Data represent mean ± SD in the number of leaves per plant **(A,B)**, leaf area (cm^2^; **C**), blade length (cm, **D**) and shoot number per plant **(E)**. Time-points with different letters are significantly different between treatments, *P* ≤ 0.05 as determined by Tukey–Kramer HSD for *H. stipulacea* and pairwise comparisons for *V. americana*.

For *H. stipulacea*, a constant increase in leaf number was observed throughout the experiment in all three salinity treatments (**Figure 4A**; one-way ANOVA with repeated measures, *P* < 0.01; **Table 1**) although plants exposed to hyposalinity (25 PSU) showed a trend of lower number of leaves throughout the experiment, compared with plants in control and hypersalinity (40 and 60 PSU, respectively). While there was no significant interaction between the effect of the salinity treatment and time on the changes in leaf number in *H. stipulacea* (one-way ANOVA with repeated measures, *P* > 0.05; **Table 1**), more senescence was seen in leaves at hyposalinities at day 33 in comparison with control plants (**Figures 3G,H**). On the other hand, *V. americana* plants exposed to hypersalinity (12 PSU) showed a significant decrease (one-way ANOVA with repeated measures, *P* < 0.05; **Table 2**) in the number of leaves compared to control plants (1 PSU) (**Figure 4B**). Furthermore, although by the end of the salinity exposure (day 33), *V. americana* plants exposed to hypersalinity possessed half the number of leaves observed in control plants (**Figure 4B**), leaf number recovered to control levels by the end of the recovery period. Significant interaction between salinity over the time of the experiment was also seen (one-way ANOVA with repeated measures, *P* < 0.05; **Table 2**). *V. americana* exposed to hypersalinity suffered large losses in above-ground biomass (**Figure 3R**), and the effects of the salinity treatment were still visible even after 3 weeks into the recovery phase (**Figure 3T**), where plants had been returned to control salinity levels.

**Table 1:** Results of one-way repeated measures ANOVA for the effects of the salinity treatment (25, 40, or 60 PSU) on different variables in *H. stipulacea*.

Variable	Effect	dF	Mean of squares	*F*	*P*-value	*n*
Leaf number	Time	1.697	855.055	14.019	0.000	5
	Time^∗^Salinity	3.395	79.254	1.299	0.303	5
	Salinity	2.000	93.195	1.621	0.238	5
Leaf area	Time	2.220	1.855	7.485	0.006	3
	Time^∗^Salinity	4.439	1.250	5.042	0.010	3
	Salinity	2.000	1.613	8.242	0.019	3
*F*_v_/*F*_m_	Time	2.899	0.001	4.836	0.007	5
	Time^∗^Salinity	5.798	0.000	1.126	0.367	5
	Salinity	2.000	0.001	1.169	0.344	5
Total chlorophyll	Time	3.325	0.100	2.600	0.061	5
	Time^∗^Salinity	9.976	0.062	1.620	0.140	5
	Salinity	3.000	0.191	1.582	0.250	5
Carotenoids	Time	3.281	0.001	2.083	0.115	5
	Time^∗^Salinity	9.844	0.001	1.911	0.077	5
	Salinity	3.000	0.004	1.253	0.338	5


*Shown are the different dependent variables, effect, degrees of freedom (df), mean of squares, F- and P-values and the number of biological replicates (n).*

**Table 2:** Results of one-way repeated measures ANOVA for the effects of the salinity treatment (1 vs. 12 PSU) on different variables in *V. americana*.

Variable	Effect	dF	Mean of squares	*F*	*P*-value	*n*
Leaf number	Time	1.486	180.234	5.021	0.034	5
	Time^∗^Salinity	1.486	353.041	9.835	0.005	5
	Salinity	1.000	775.557	11.250	0.010	5
Blade length	Time	1.184	44.613	28.970	0.001	4
	Time^∗^Salinity	1.184	41.177	26.739	0.001	4
	Salinity	1.000	42.875	8.258	0.028	4
Shoot number	Time	1.550	12.637	7.649	0.014	4
	Time^∗^Salinity	1.545	3.788	2.293	0.160	4
	Salinity	1.000	8.383	6.717	0.041	4
*F*_v_/*F*_m_	Time	2.519	0.001	0.717	0.534	4
	Time^∗^Salinity	2.519	0.003	3.076	0.066	4
	Salinity	1.000	0.000	0.359	0.571	4
Total chlorophyll	Time	2.123	281.552	19.599	0.000	5
	Time^∗^Salinity	2.123	48.893	3.403	0.055	5
	Salinity	1.000	17.503	11.410	0.010	5
Carotenoids	Time	2.439	16.052	23.370	0.000	5
	Time^∗^Salinity	2.439	2.145	3.123	0.058	5
	Salinity	1.000	2.009	27.76	0.001	5


*Shown are the different dependent variables, effect, degrees of freedom (df), mean of squares, F-and P-values and the number of biological replicates (n).*

### Leaf Area and Blade Length

Exposure to hypersalinity resulted in a large decrease in leaf area (**Figure 4C**) in *H. stipulacea* (one-way ANOVA with repeated measures, *P* < 0.05; **Table 1**). A trend toward reduced leaf area was also observed in response to hyposalinity, but this decrease was smaller, slower and not statistically significant (one-way ANOVA with repeated measures followed by Tukey test, *P* > 0.05; **Table 1**). However, leaf area in plants exposed to hyposalinity continued to drop even after plants were returned to control salt levels until there was a significant leaf area reduction compared to control plants by day 60 (one-way ANOVA with repeated measures followed by *Post hoc* Tukey test, *P* < 0.05). On the other hand, leaf area in *H. stipulacea* plants exposed to hypersalinity stopped decreasing once plants were returned to control salt levels although leaf area did not recover to that of control plants at least over the period allowed for recovery in this experiment. The interaction between the salinity treatment and time was also significant (one-way ANOVA with repeated measures, *P* < 0.05; **Table 1**).

In *V. americana*, there was a significant difference over time in blade length between the control and stressed plants immediately after the plants were exposed to high salinity (one-way ANOVA with repeated measures, *P* < 0.05; **Table 2** and **Figure 4D**). However, most of the leaves that were marked for blade length measurement were lost during the recovery phase and therefore no measurements could be taken for the last time point in the stressed *V. americana* plants. The increase in *V. americana* leaf number during the recovery phase was due to new leaf production (**Figure 4B**).

A significant decrease was also observed in shoot number (one-way ANOVA with repeated measures, *P* < 0.05; **Table 2**) in stressed *V. americana* plants in comparison to the plants at control salinity (**Figure 4E**). Pairwise comparisons showed significant differences at days 26 and 33 (one-way ANOVA with repeated measures followed by pairwise comparisons, *P* < 0.05). However, there was no significant interaction of salinity and time (one-way ANOVA with repeated measures, *P* > 0.05; **Table 2**). During recovery, *V. americana* plants produced new shoots and at day 60 there was no significant difference between control plants and plants that were exposed to hypersalinity (one-way ANOVA with repeated measures followed by pairwise comparisons; *P* > 0.05).

### *H. stipulacea* Exhibits Stress-Resilient Photosynthetic Capacity

**FIGURE 5:**
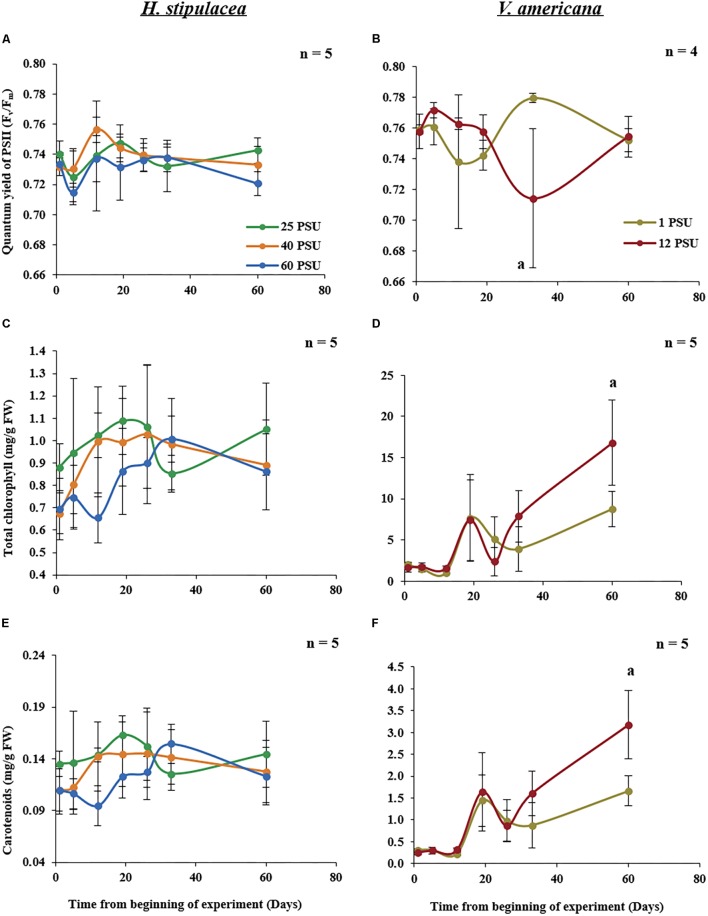
Photo-physiological parameters measured in *H. stipulacea* (Left; **A,C,E**) and *V. americana* (Right **B,D,F**) throughout the experiment. Data represent mean ± SD in dark-adapted quantum yield of PSII (*F*_v_/*F*_m_; **A,B**), total chlorophyll (chlorophyll a+b; **C,D**) and carotenoid **(E,F)** concentrations. Time-points with different letters are significantly different between treatments, *P* ≤ 0.05 as determined by Tukey–Kramer HSD for *H. stipulacea* and pairwise comparisons for *V. americana*.

Measurements of chlorophyll fluorescence parameters can provide information about photochemistry that is driving photosynthesis (Maxwell and Johnson, 2000). In *H. stipulacea*, *F*_v_/*F*_m_, which evaluates the maximum efficiency of photosystem II (PSII; Maxwell and Johnson, 2000), showed no significant interaction between salinity and time (one-way ANOVA with repeated measures, *P* > 0.05; **Table 1**) throughout the experiment (**Figure 5A**). There were also no significant differences in total leaf chlorophyll content (**Figure 5C**), carotenoid levels (**Figure 5E**) or chlorophyll *a* and chlorophyll *b* (data not shown) between plants under control and salinity treatments over the time of the experiment (one-way ANOVA with repeated measures, *P* > 0.05; **Table 1**). However, in *V. americana*, *F*_v_/*F*_m_ exhibited a strong trend (one-way ANOVA with repeated measures, *P* = 0.0534; **Table 2**) for differences between treatments over time. However, this lack of significant difference between control and salt-stressed plants lasted only until day 33 by which time *F*_v_/*F*_m_ was significantly reduced (one-way ANOVA with repeated measures followed by *Post hoc* Tukey test, *P* < 0.05) in plants exposed to hypersalinity (**Figure 5B**). After return to control salinity, the stressed plants were able to recover and there was no significant difference between the control and stressed plants at day 60. While total chlorophyll and carotenoid contents for *V. americana* plants also displayed no significant difference between treatments over time (one-way ANOVA with repeated measures, *P* > 0.05; **Table 2**), there were strong trends that indicated some differential effect (**Table 2**) between control and salt-stressed *V. americana* plants (**Figure 5D**). This is suggested by differences in chlorophyll and carotenoid contents between the treatments shown toward the end of the experiment, after the return of salt-stressed plants to control salt conditions, where both total chlorophyll (**Figure 5D**) and carotenoid (**Figure 5F**) levels increased significantly above that of control plants (one-way ANOVA, followed by Pairwise comparisons, *P* < 0.05; **Figure 5F**).

### Changes in Salinity Cause a Greater Effect on the Biomass and C/N Ratios of *H. stipulacea* Than *V. americana*

**FIGURE 6:**
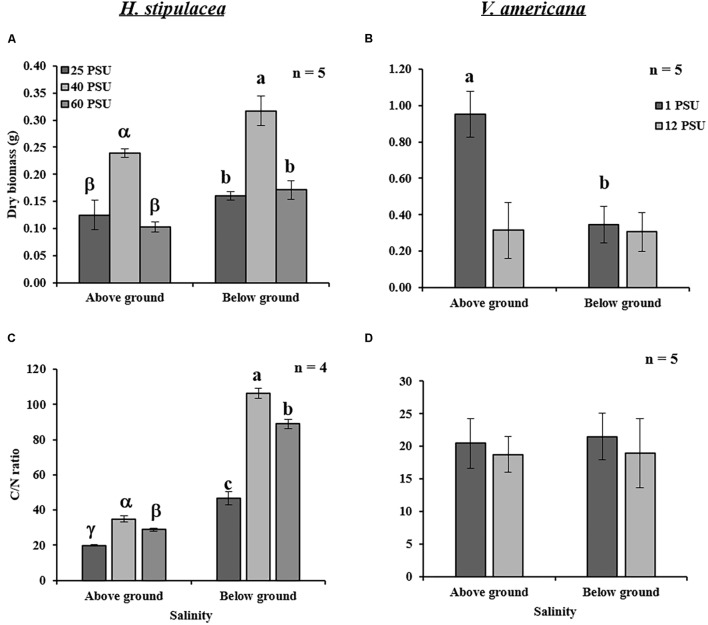
Effects of salinity treatments (see legend of **Figure 5** for details) on the above- and below-ground dry biomass and C/N ratios in *H. stipulacea* (Left; **A,C**) and *V. americana* (Right; **B,D**) measured the end of the experiment (day 60 only). Data represent mean ± SD. Bars with different letters are significantly different between treatments, *P* ≤ 0.05 as determined by Tukey–Kramer HSD for *H. stipulacea* and pairwise comparisons for *V. americana*.

Biomass and C/N ratios of *H. stipulacea* and *V. americana* were determined from plants harvested at day 60 (27 days into the recovery period). A significant decrease (one-way ANOVA, *P* < 0.05; **Table 3**) in the biomass of both below- and above-ground tissues from *H. stipulacea* plants previously exposed to hypo or hypersalinity was observed compared to plants that were maintained during the entire experimental period in control salinities (**Figure 6A**). This finding indicates a long-lasting impact of the salinity treatment on *H. stipulacea* plants. A long-term effect of salinity on *V. americana* biomass was also observed but only in above-ground tissues (**Figure 6B**; one-way ANOVA, *P* < 0.05; **Table 4**). There was no significant difference (one-way ANOVA, *P* > 0.05; **Table 4**) in biomass of below-ground *V. americana* tissues between control and salt-treated plants.

**Table 3:** Results of one-way ANOVA for the effects of the salinity treatment (25, 40, or 60 PSU) on different variables in *H. stipulacea* at day 60.

Variable	Effect	dF	Mean of squares	*F*	*P*-value	*n*
C/N above-ground	Salinity	2.000	82.190	14.692	0.001	3
C/N below-ground	Salinity	2.000	61.642	7.346	0.024	3
Biomass above-ground	Salinity	2.000	0.018	5.260	0.023	5
Biomass below-ground	Salinity	2.000	0.034	20.966	0.000	5


*Shown are the different dependent variables, effect, degrees of freedom (df), mean of squares, F- and P-values and the number of biological replicates (n).*

**Table 4:** Results of one-way ANOVA for the effects of the salinity treatment (1 vs. 12 PSU) on different variables in *V. americana* at day 60.

Variable	Effect	dF	Mean of squares	*F*	*P*-value	*n*
C/N above-ground	Salinity	1.000	7.200	0.652	0.443	5
C/N below-ground	Salinity	1.000	15.996	0.775	0.404	5
Biomass above-ground	Salinity	1.000	1.020	51.752	0.000	5
Biomass below-ground	Salinity	1.000	0.004	0.362	0.564	5


*Shown are the different dependent variables, effect, degrees of freedom (df), mean of squares, F- and P-values and the number of biological replicates (n).*

C/N ratios of *H. stipulacea* above- and below-ground tissues exposed to both hypo and hypersalinity exhibited a significant reduction at the end of the experiment (one-way ANOVA, *P* < 0.05; **Table 3**) compared to control plants (**Figure 6C**). However, no differences (one-way ANOVA, *P* < 0.05; **Table 4**) in *V. americana* C/N ratios between control and salt-treated plants were observed in either above- or below-ground tissues (**Figure 6D**).

## Discussion

In terms of their ability to withstand marine salinities, seagrasses are defined as halophytes (Zhu, 2001; Touchette, 2007; Munns and Tester, 2008). This is probably even more so for the tropical *H. stipulacea* since it is dominant in the northern Red Sea, where salinity is rarely lower than 40–41 PSU (Katz et al., 2015). While it is obvious that *H. stipulacea* and *V. americana* are very different plants that exist in very different habitats and in particular, different salt environments, they are genetically very close (Les et al., 1997, 2006). Both these species are capable of extensive clonal growth (Lovett-Doust and Laporte, 1991), and both have a relatively wide, although not overlapping, salt range. Here, we are not comparing salt tolerance *per se* between the two species but comparing the responses of each plant to changes in salinity. In this context, it is important to note that the transcriptome and metabolome of the halophytic *Arabidopsis* relative, *Eutrema salsugineum* exhibit a much lower global response to salt stress than salt-sensitive *Arabidopsis* (Gong et al., 2005; Lugan et al., 2010; Kazachkova et al., 2013). However, at higher salinity levels than is required for *Arabidopsis* to respond, stress-mediated induction of the *E. salsugineum* transcriptome does occur suggesting that the stress-sensitive and stress-tolerant species possess different sensitivities to salt (Gong et al., 2005; Amtmann, 2009). A similar situation could occur between *V. americana* and *H. stipulacea* as the seagrass only exhibits an effect of salt on growth at a far higher salt level than its freshwater relative (**Figures 3**, **4**).

Most seagrasses are thought to be sensitive to hypersalinity (Ogata and Matsui, 1965; Biebl and McRoy, 1971; Zieman, 1975; Adams and Bate, 1994; Kamermans et al., 1999; Van Katwijk et al., 1999). While there are a few studies on the effect of temperature and light on *H. stipulacea* (Angel et al., 1995; Schwarz and Hellblom, 2002; Sharon et al., 2009, 2011), this is, to the best of our knowledge, the first paper exploring the salinity range and effects of salinity on the physiology and growth of this euryhaline seagrass species.

### Leaf Size Modulation – An Important Mechanism to Cope With Salinity in *H. stipulacea*

Among other traits such as ion homeostasis, photosynthesis, yield components and senescence, plant growth is an important trait associated with salt stress (Negrão et al., 2017). Plants reduce their growth rate immediately after the onset of stress and to begin to conserve and distribute their resources as needed (Skirycz and Inzé, 2010). Several reports on terrestrial plants suggest a reduction in leaf area as a general response to salinity stress (Marcelis and Van Hooijdonk, 1999; Wang and Nii, 2000; Maggio et al., 2007). Our results support this general response to stress – *H. stipulacea* suffered reductions in leaf area when exposed to hyper or hyposalinity (**Figure 4C**), with hyposalinity also causing a trend toward reduction in leaf number (**Figure 4A**). Previous studies showed that *H. stipulacea* modified leaf size in response to varying temperature (Procaccini et al., 1999), light levels (Schwarz and Hellblom, 2002; Mejia et al., 2016; Rotini et al., 2017) and hydrodynamics (Procaccini et al., 1999; Mejia et al., 2016). Hence, leaf size modulation may be an important mechanism in *H. stipulacea* to cope not only with salinity but with other abiotic stresses as well. It might be worth noting that under hyposalinity, leaf area was not very different from control plants until after day 33. This could be considered as a late response or damage, at least in comparison to *H. stipulacea*’s response to hypersalinity.

In contrast, *V. americana* showed a significant reduction in the number of leaves, number of shoots/plant and blade length (**Figures 4B,D,E**). In the case of leaf and shoot number, *V. americana* seemed to only show an effect of hypersaline stress 3 weeks after stress induction. These results are in agreement with other studies suggesting a similar effect of salinity on blade length, shoot number and leaf number in this freshwater species (Bourn, 1932, 1934; Haller et al., 1974; Frazer et al., 2006). Boustany et al. (2010) showed hardly any negative effects on *V. americana* plants of 3 weeks exposure to 18 PSU but nearly complete mortality after 10 weeks of exposure which also complements other reports including Davis and Brinson (1976), Staver (1986), and French and Moore (2003). In our study, during the recovery period, there was an increase in leaf and shoot number comparable to the levels of the plants grown in control conditions. This recovery was similar to the recovery observed by Doering et al. (2001), that summarized recovery of *V. americana* plants in two stages: (i) the allocation of energy to the production of new blades on individual shoots; (ii) the production of new shoots with blades. Though the recovery period used by Doering et al. (2001) was over 2 months (compared with our 1-month recovery period), our results demonstrating increases in shoot and leaf number, confirm similar recovery patterns. It is interesting to note that *V. americana* plants responded immediately to hypersalinity by slowing down the growth of existing leaves (**Figure 4D**). There were no measurements for the last time point after recovery because the leaves marked for measurements of blade length were lost (**Figure 3T**). This could be due to *V. americana* accumulating salt in older leaves and then shedding them, which is a known plant response to salinity (Munns and Tester, 2008), and it will thus be important to perform detailed measurement of ion concentrations in tissues of both *V. americana* and *H. stipulacea*.

### *H. stipulacea* Photochemical Characteristics Are Resilient to Hypo and Hypersalinity

Quantum efficiency of photosystem II (*F*_v_/*F*_m_) has been used an as an indicator of stress in seagrasses (Biber et al., 2005; Massa et al., 2009; Winters et al., 2011; Dooley et al., 2013) including salinity stress (Ralph, 1998; Fernández-Torquemada and Sánchez-Lizaso, 2005; Koch et al., 2007). Studies on other seagrasses indicate that *F*_v_/*F*_m_ is negatively affected by exposure to salinity stress over time (Ralph, 1998; Kahn and Durako, 2006; Pagès et al., 2010). *H. stipulacea* was reported to have ‘plastic’ photosynthetic capabilities at varying irradiances, and even showing an alteration in its PSI:PSII ratio (Sharon et al., 2009, 2011). Previous studies on *H. stipulacea* have also shown that enzymes involved in photosynthesis, RuBP and PEPcase, and located in the epidermal tissue, were active even under higher salinity conditions (Beer et al., 1980). *H. stipulacea* also displayed lower epidermal concentrations of Na^+^ and Cl^-^ ions when compared to the exterior and other tissues (Beer et al., 1980). This might be one of the mechanisms by which *H. stipulacea* maintains photosynthetic capacity throughout the experiment in both hypo and hypersalinity conditions (**Figure 5A**). It is well-known that to maintain photosynthetic efficiency, plants change the concentrations of their photosynthetic pigments in response to changes in water quality and light regimes (Campbell et al., 2003; Ralph et al., 2007). Indeed, there were changes in number and ultrastructure of chloroplasts in *H. stipulacea* under high light conditions (Beer et al., 1980). In contrast to other seagrasses (Kahn and Durako, 2006; Pagès et al., 2010), our results show that there was no significant decrease or increase in the dark-adapted quantum yield (*F*_v_/*F*_m_) and chlorophyll content (**Figures 5A,C**) in response to salinity stress, indicating a rather stable photosynthetic capacity. In contrast, *V. americana* showed a reduction in *F*_v_/*F*_m_ values during the experiment, but this took time and was evident only after day 19, which might be an indication of accumulative damage to the PSII reaction centers (Maxwell and Johnson, 2000). However, in terms of *F*_v_/*F*_m_, the *V. americana* plants demonstrated full recovery when returned to control salinity conditions as seen in the measurements at day 60 (**Figure 5B**). It is worth mentioning that although *F*_v_/*F*_m_ measurements are widely used and are reliable diagnostic indicators of photophysiological stress (Murchie and Lawson, 2013) and photoinhibition (Winters et al., 2003), non-photochemical quenching, and effective quantum yield measurements (Ralph, 1998) will give us a more complete picture of the photosynthetic properties of *H. stipulacea* and *V. americana*. In *V. americana* plants, our results also show an increase in total chlorophyll and carotenoid content under hypersalinity (**Figures 5D,F**). This is in contrast to other studies which showed a reduction in chlorophyll content at higher salinities suggesting effects on the photochemical efficiency of the plants (Doering et al., 1999; French and Moore, 2003; Boustany et al., 2015). The increase in chlorophyll and carotenoid content observed in *V. americana* may be due to an increase in the number of chloroplasts as seen in other studies (Aldesuquy and Gaber, 1993; Misra et al., 1997). However, further studies are required to confirm these observations.

### *H. stipulacea* Might Mobilize Stored Reserves From Below-Ground Tissues to Support Production of New Leaves

For *H. stipulacea*, measurements made after 27 days of recovery (day 60), showed significant hyper and hyposalinity-mediated reduction in above- and below-ground biomass (**Figure 6A**). Loss of above-ground biomass may be seen as a response to salinity by *H. stipulacea* in an attempt to survive by producing new leaves but with reduced leaf area. Exposure to both hypo and hypersalinity treatments caused a long-term change in the biomass of the rhizomes, which are also associated with storage of carbohydrates (e.g., sucrose; Gu et al., 2012). Indeed, stress was shown to reduce underground biomass in the temperate seagrass *Z. marina*. In *Z. marina*, light limitation suppressed production of new roots, led to a reduction of sucrose reserves, and caused a decrease in growth rate with increased sucrose synthase activity in leaf tissues toward the end of the stress (light limitation) period (Alcoverro et al., 1999). Sucrose synthase is a key enzyme involved in degradation of sucrose and increased activity of the enzyme indicated that sucrose was being used for the synthesis of more effective and compatible solutes (Touchette, 2007). It was previously shown that light or carbon limitation mobilizes stored reserves to support shoot or leaf proliferation at the expense of below-ground growth (Zimmerman and Alberte, 1996; Clabby and Osborne, 1997; Zimmerman et al., 1997). This ability, if present in *H. stipulacea*, needs to be confirmed through further studies including accumulation/depletion of specific amino acids or osmolytes in response to changing salinity.

The long-term loss of underground carbon storage was also evident by the C/N ratios measured at the end of the recovery period (**Figures 6C,D**). *H. stipulacea* plants that were exposed to high or low salinities showed significant decreases in the above- and below-ground C/N ratios even at day 60, 27 days after they were returned to control salinity levels (**Figure 6C**). There is a strong correlation between the C/N ratio of seagrasses with N concentration within the plant (Duarte, 1992). *H. stipulacea* showed higher C/N ratios in roots and rhizomes relative to leaves in plants at depths shallower than 24 m mainly due to lower nitrogen concentrations (Schwarz and Hellblom, 2002). In agreement with these results, our results showed that below-ground tissues exhibited a lower N concentration than the above-ground tissues under control and stressed conditions (**Figure 6C**). The reduction in C/N ratio observed under hypo and hypersalinities was due to an increase in N content (Supplementary Table S1) in comparison to control plants. These results are in line with known studies showing an increase in tissue nitrogen content and certain amino acids at hypersaline conditions (Pulich, 1986; Twilley and Barko,1990).

While studies have shown a significant decrease in both below- and above-ground biomass in *V. americana* as a response to increased salinities (Frazer et al., 2006; Boustany et al., 2015), only a few studies have shown that the roots might be more tolerant to increased salinity than shoots (Doering et al., 2001; Boustany et al., 2010). Boustany et al. (2010) reported that when *V. americana* plants were exposed to hypersalinity (8 PSU), there was an increase in the root:shoot ratio. Furthermore, at 18 PSU the plants lost all the above-ground biomass but 20% of the roots were able to regrow when the plants were returned to control conditions (Boustany et al., 2010). Doering et al. (2001) showed an 80% reduction in above-ground biomass in *V. americana* plants exposed to 18 PSU salinity, which recovered after 115 days of growth back in control salinity conditions. In the study presented here, exposure to hypersalinity of 12 PSU caused a 60% reduction in the above-ground *V. americana* biomass, even after recovery (**Figure 6B**). Although the duration of our experiment was not as long as the other studies, the effects of salinity on the shoots showed similar results, confirming that roots were more tolerant to hypersalinity than shoots.

Surprisingly, C/N ratio measurements in *V. americana* did not show any significant change between the control and treated plants (Supplementary Table S2). This might have been due to recovery of the plants, as shown by the recovery of leaf number during the period from days 33 to 60 (**Figure 4B**). Similarly, Boustany et al. (2015) observed that recovery from salinity begins as soon as control salinity conditions are restored. Therefore, the possibility that C/N ratio in *V. americana* was affected by hypersalinity cannot be ruled out.

Although the emphasis in this study was on physiology, the results shown here also have important ecological implications. While *H. stipulacea* is native to the Red Sea and the Indian Ocean, it is invasive in the Mediterranean (Gambi et al., 2009) and the Caribbean (Steiner and Willette, 2014). With the increases in brine discharges in the Mediterranean, Red Sea and Persian Gulf (Bashitialshaaer et al., 2011) and with the Mediterranean Sea undergoing a process of ‘tropicalization’ (Bianchi and Morri, 2003; Borghini et al., 2014), it is becoming warmer and saltier (changes of 0.12°C ± 0.07 year^-1^ and 0.008 ± 0.006 year^-1^ in water temperatures and salinities, respectively in the Eastern Mediterranean; Ozer et al., 2017). This process will occur even faster after the recent doubling of the capacity of the Suez Canal (“Double Trouble”; Galil et al., 2015, 2017). Thus, the potential threat to local Mediterranean biodiversity posed by *H. stipulacea* is considered serious. Indeed, *H. stipulacea* has been included in the “100 Worst Invasive Alien Species in the Mediterranean” (Streftaris and Zenetos, 2006). This concern is even more warranted considering the alarming studies from the Caribbean showing that *H. stipulacea* is actually displacing local seagrass species (Steiner and Willette, 2014), and the studies from the Mediterranean showing vast declines in the local slow-growing *P. oceanica* (Marba and Duarte, 2010; Jordà et al., 2012).

Similar to the tolerance to increased salinities, the tolerance to reduced salinities might provide *H. stipulacea* an advantage in situations of terrestrial freshwater run-offs when local salinity levels temporarily drop (e.g., Katz et al., 2015; Winters et al., 2017).

The current study demonstrates that *H. stipulacea* is quite capable of tolerating, at least in the short-term (3–4 weeks), both increased (60 PSU) and decreased salinities (25 PSU). While we have no information about other seagrass species from this region, if indeed *H. stipulacea* has a wider salinity tolerance than other local species, it might have an advantage in the coming future compared with other seagrass species that might tolerate a much narrower range of salinity changes. We therefore hypothesize that tolerance to a wide range of salinities (both hypo and hypersalinities) could provide *H. stipulacea* with an advantage compared with other seagrass species, and might explain some of its opportunistic and invasive character. We might be seeing much more *H. stipulacea* in the impending future.

## Conclusions and Perspectives

How do these closely related species – *H. stipulacea* and *V. americana* inhabit environments with such different salinities? Answering this question would help us not only in understanding the ability of *H. stipulacea* to survive in new environments (Dittami et al., 2017) and predict extension of this invasive species to other seas but also aid in elucidating salt tolerance mechanisms in seagrasses and plants in general.

Our results demonstrate that *H. stipulacea* has a remarkable tolerance to hyper and hyposalinity, and it is likely that *H. stipulacea* possesses salinity tolerance mechanisms that are absent in its close freshwater relative. The most visible differences in salt tolerance between the two species are leaf size modulation and the ability to produce new shoots probably at the expense of the below-ground tissue in *H. stipulacea*. Clearly, *H. stipulacea* is able to maintain its photosynthetic capability under both hyper and hyposalinity. This resilience to changing salinity may also be an important trait explaining the invasive nature of this species in the Caribbean Sea. Even though *H. stipulacea* does not exhibit much response to changing salinities in terms of growth and photosynthesis, the effects of hyper and hyposalinity in this seagrass cannot be dismissed considering the salt-mediated effects on biomass and C/N ratios. Contrasting responses to hypersalinity between *H. stipulacea* and *V. americana* are most evident just before and after the recovery phase; during this shift from hypersalinity back to control salinity levels, *V. americana* displays recovery of all growth and photochemical measurements to pre-stress values. The roots/below-ground tissues of *V. americana* are more tolerant to changing salinities in comparison with *H. stipulacea*. On-going work has moved on to molecular profiling in both these closely related species and holds great potential in separating molecular traits associated with adaption to an aquatic lifestyle (found in both *H. stipulacea* and *V. americana*) and those specifically associated with adaptation to high levels of salinity associated with the marine environment (found only in *H. stipulacea*). With recent studies on the microbiome of *H. stipulacea* (Mejia et al., 2016; Rotini et al., 2017) it is increasingly believed that plant–microbe interactions play an important role in plant adaptation to new environments. It would be interesting to study how much of *H. stipulacea*’s tolerance to salinity may be attributed to its associated microbes. More studies including accumulation of osmolytes and compartmentalization of Na^+^ ions in both these plant species would reveal interesting details regarding plant tolerance mechanisms. One way of comprehending ecological traits such as salinity is to combine phenotypic and physiological assessments with transcriptomic and their equivalent metabolic pathways (Exadactylos, 2015). With the emergence of molecular profiling and omics techniques in seagrass biology (Procaccini et al., 2007; Davey et al., 2016; Lee et al., 2016; Olsen et al., 2016), recent studies have focused on the effects of light, increased water temperature, salinity, high CO_2_ levels at the transcriptomic level in seagrasses. These studies are revealing new insights into mechanisms adapted by seagrasses to survive under various abiotic stresses (Franssen et al., 2011; Kong et al., 2014; Piro et al., 2015a,b; Salo et al., 2015; Marín-Guirao et al., 2017; Ruocco et al., 2017). Comparisons of the transcriptome and the metabolome of *H. stipulacea* and *V. americana* might reveal more about salinity tolerance mechanisms present in *H. stipulacea*.

## Author Contributions

GW, SB, and MAO conceived and designed the experiments. GW performed the sampling of plants and contributed to the preparation of the samples. MAO performed the physiological measurements. MAO, GW, and SB wrote the manuscript.

## Conflict of Interest Statement

The authors declare that the research was conducted in the absence of any commercial or financial relationships that could be construed as a potential conflict of interest.

## Footnotes

1 http://www.iui-eilat.ac.il/Research/NMPmeteodata.aspx

2 https://www.osram.com

3 https://www.redseafish.com/red-sea-salts/red-sea-salt/

4 http://www.ofra-aqua.co.il
