# Supplementary material for: The Tropical Invasive Seagrass, Halophila stipulacea, Has a Superior Ability to Tolerate Dynamic Changes in Salinity Levels Compared to Its Freshwater Relative, Vallisneria americana
Source: Front Plant Sci. 2018 Jul 4;9:950. doi: 10.3389/fpls.2018.00950 (PMC6040085; doi:10.3389/fpls.2018.00950)
Supplement: Supplementary file 1 [file Table_1.DOCX]

**Supplementary Table S1**. Nitrogen and Carbon percentages by dry weight (DW) obtained after elemental analysis after day 60 in *H. stipulacea*. Values are averages of 10 measurements. Control for *H. stipulacea* is 40 PSU.

|  | **25 PSU** | | **40 PSU** | | **60 PSU** | |
| --- | --- | --- | --- | --- | --- | --- |
| **Tissue** | **Nitrogen (%)** | **Carbon (%)** | **Nitrogen (%)** | **Carbon (%)** | **Nitrogen (%)** | **Carbon (%)** |
| Above ground | 1.48298974 | 29.59322433 | 0.848284829 | 29.56688271 | 1.031211078 | 29.80960159 |
| Below ground | 0.611032 | 28.47904739 | 0.281605368 | 29.99956398 | 0.334132981 | 29.71052818 |
