# Supplementary material for: The Tropical Invasive Seagrass, Halophila stipulacea, Has a Superior Ability to Tolerate Dynamic Changes in Salinity Levels Compared to Its Freshwater Relative, Vallisneria americana
Source: Front Plant Sci. 2018 Jul 4;9:950. doi: 10.3389/fpls.2018.00950 (PMC6040085; doi:10.3389/fpls.2018.00950)
Supplement: Supplementary file 2 [file Table_2.DOCX]

**Supplementary Table S2**. Nitrogen and Carbon percentages by dry weight (DW) obtained after elemental analysis after day 60 in *V. americana*. Values are averages of 10 measurements. Control for *V. americana* is 1 PSU.

|  | **1 PSU** | | **12 PSU** | |
| --- | --- | --- | --- | --- |
| **Tissue** | **Nitrogen (%)** | **Carbon (%)** | **Nitrogen (%)** | **Carbon (%)** |
| Above ground | 1.54604 | 30.64195951 | 1.77679 | 32.68867963 |
| Below ground | 1.53968 | 34.63516006 | 1.60025 | 34.55540403 |
