# Supplementary figures and images for: The Tropical Invasive Seagrass, Halophila stipulacea, Has a Superior Ability to Tolerate Dynamic Changes in Salinity Levels Compared to Its Freshwater Relative, Vallisneria americana
Source: Front Plant Sci. 2018 Jul 4;9:950. doi: 10.3389/fpls.2018.00950 (PMC6040085; doi:10.3389/fpls.2018.00950)

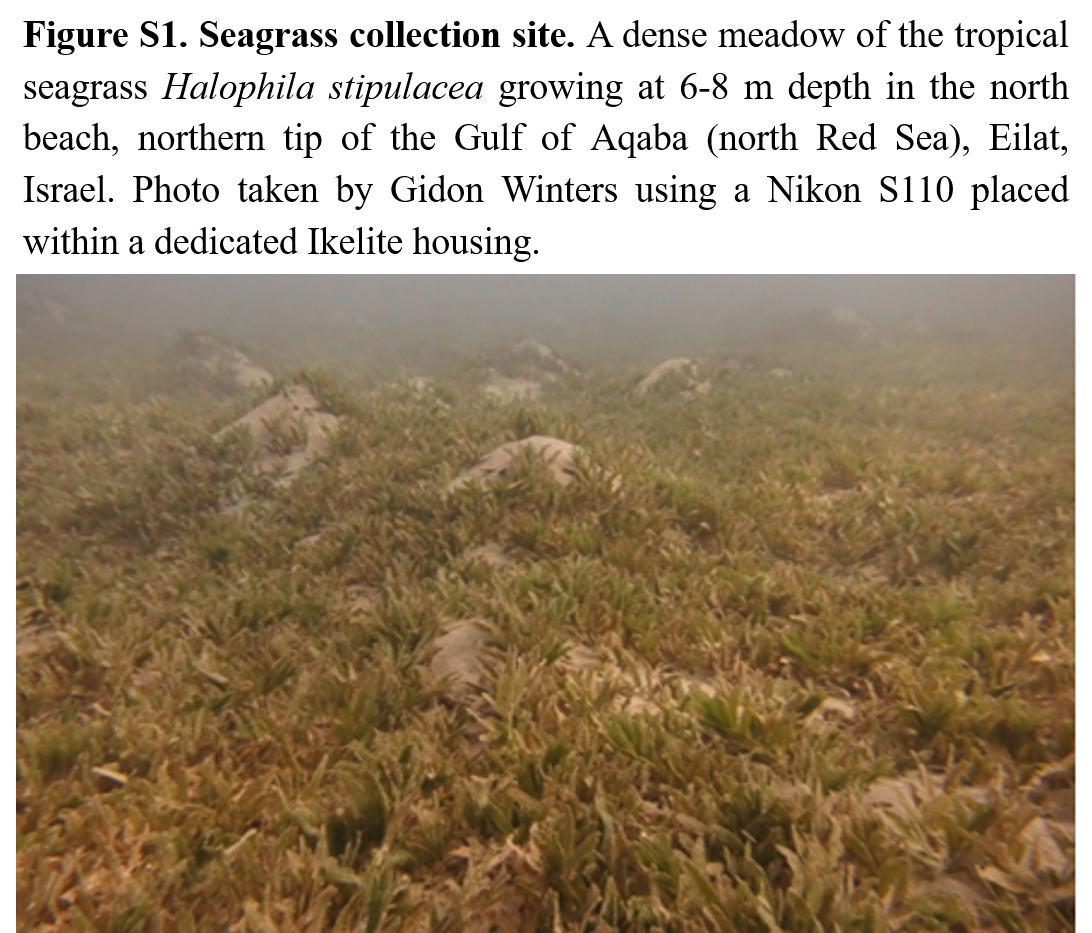

Supplement: Supplementary file 3 [file Image_1.TIF]

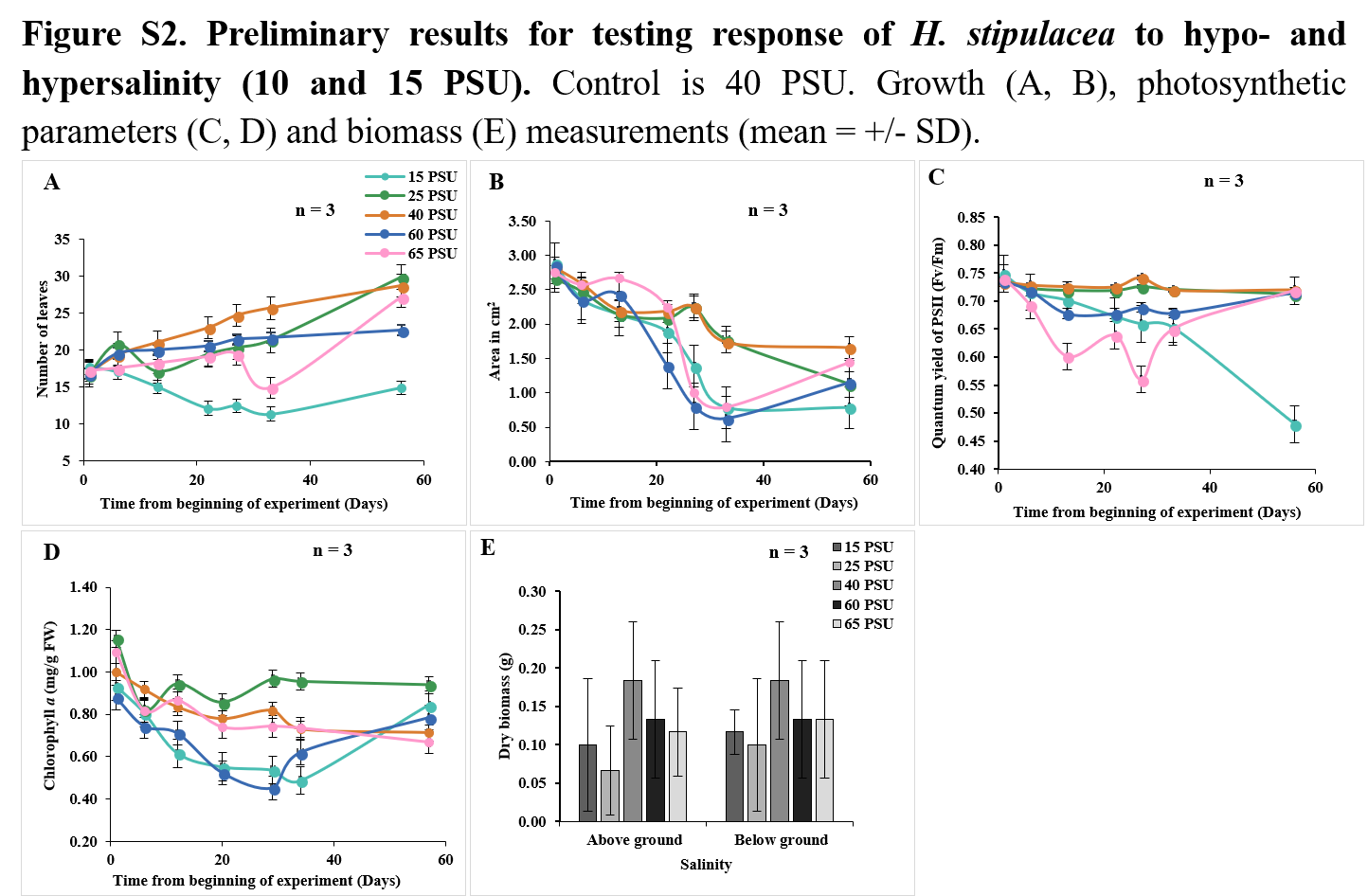

Supplement: Supplementary file 4 [file Image_2.TIF]

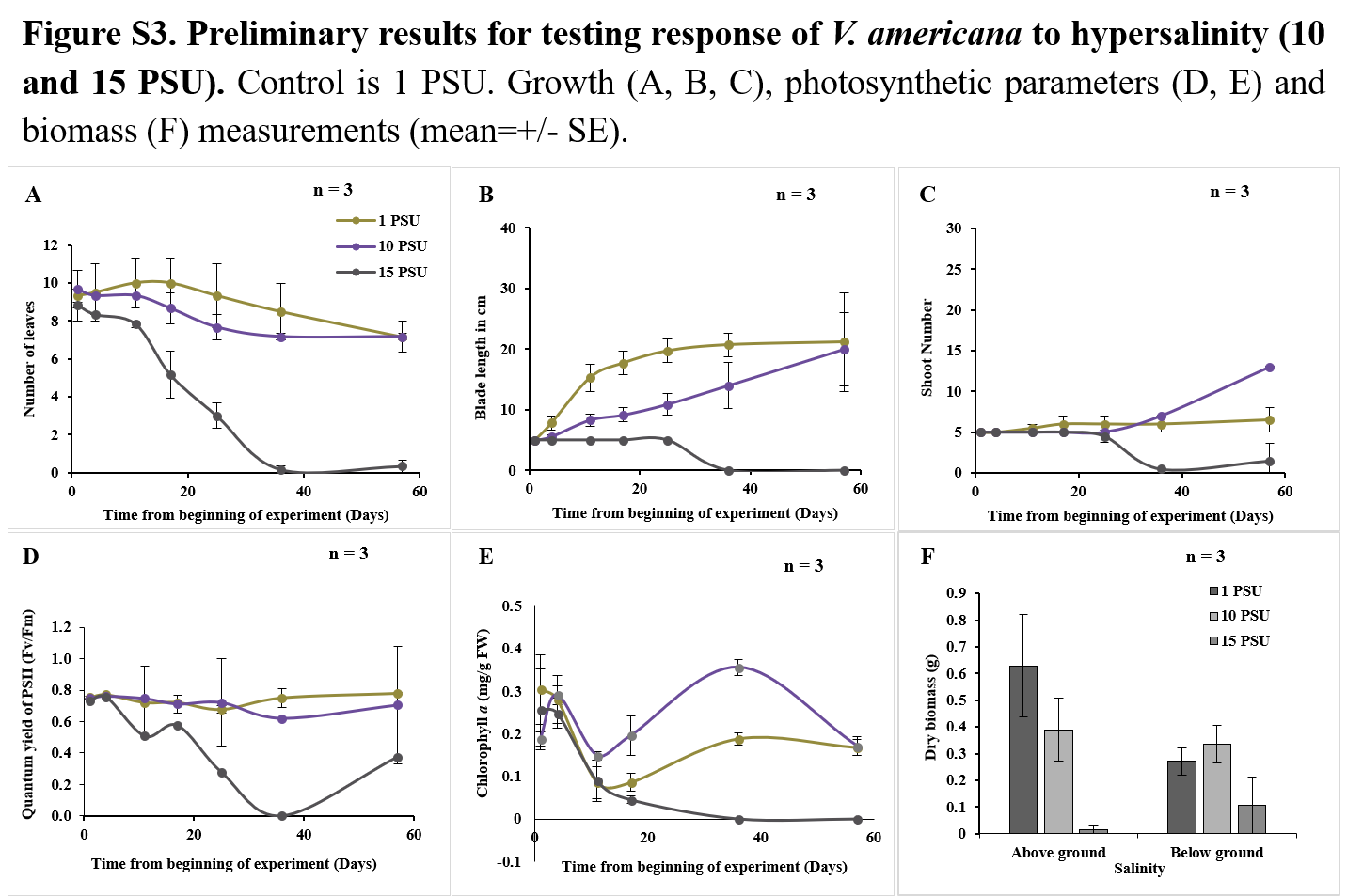

Supplement: Supplementary file 5 [file Image_3.TIF]
